# Budesonide MMX for the Induction of Remission of Mild to Moderate Ulcerative Colitis: A Pooled Safety Analysis

**DOI:** 10.1093/ecco-jcc/jjv101

**Published:** 2015-06-20

**Authors:** Gary R. Lichtenstein, Simon Travis, Silvio Danese, Geert D’Haens, Luigi Moro, Richard Jones, Michael Huang, E. David Ballard, Robert Bagin, Yun Hardiman, Raul Collazo, William J. Sandborn

**Affiliations:** ^a^Division of Gastroenterology, Department of Medicine, University of Pennsylvania, Philadelphia, PA, USA; ^b^Translational Gastroenterology Unit, Oxford University Hospitals, Oxford, UK; ^c^Instituto Clinico Humanitas, Milan, Italy; ^d^Academic Medical Center, Amsterdam, The Netherlands; ^e^Cosmo Technologies Ltd, Dublin, Ireland; ^f^Santarus, Inc., San Diego, CA, USA; ^g^University of California San Diego, La Jolla, CA, USA

**Keywords:** Budesonide MMX, ulcerative colitis, safety, tolerability

## Abstract

**Background and aims:**

Cumulative safety and tolerability of budesonide MMX, a once-daily oral corticosteroid for inducing mild to moderate ulcerative colitis remission, was examined.

**Methods:**

Data from three randomized, double-blind, placebo-controlled, phase II or III studies [budesonide MMX 9mg, 6mg, or 3mg for 8 weeks]; one phase II study [randomisation to budesonide MMX 9mg or placebo for 4 weeks, then open-label budesonide MMX 9mg for 4 weeks]; and one open-label study [budesonide MMX 9mg for 8 weeks] were pooled.

**Results:**

Patients randomised to budesonide MMX 9mg [*n* = 288], 6mg [*n* = 254], or placebo [*n* = 293] had similar rates of adverse events [AEs] [27.1%, 24.8%, and 23.9%, respectively] and serious AEs [2.4%, 2.0%, and 2.7%, respectively]; treatment-related AEs and serious AEs were reported by 11.8% and 13.5%, and 5.9% and 2.2%, respectively, of patients receiving budesonide MMX 3mg [*n* = 17] or open-label budesonide MMX 9mg [*n* = 89]. Mean morning plasma cortisol concentrations were normal from baseline to final visit across randomised groups; in patients receiving open-label budesonide, mean cortisol concentration was 129.9 nmol/l after 4 weeks, returning to normal concentrations at final visit. Budesonide MMX was not associated with an overall increased risk for glucocorticoid-related adverse effects.

**Conclusions:**

Budesonide MMX 9mg was associated with normal mean cortisol concentrations at final visit and an AE incidence comparable to placebo. Overall, budesonide MMX was safe and well tolerated for inducing remission of patients with mild to moderate ulcerative colitis.

## 1. Introduction

The prevalence of ulcerative colitis [UC] has been noted to be up to 0.51% of the population in Europe, 0.17% in Asia and the Middle East, and up to 0.25% in North America.^1^ UC is associated with a high degree of healthcare utilisation and burden of disease. From 1998 to 2004, there was a 3% annual increase in hospitalisation rates for UC,^2^ and direct medical costs were estimated to be > US $2 billion in 2004.^3^ Current treatment guidelines, edited by the professional bodies or associations up to 2012, recommend 5-aminosalicylic acid [5-ASA] as first-line therapy for the induction of remission in patients with mild to moderate UC.^4,5^ Systemic corticosteroids are usually required as the disease progresses, but are associated with safety concerns, including adrenal suppression, infections, ophthalmic effects [eg cataracts, glaucoma], and mood changes.^4,6^

The potent, second-generation corticosteroid budesonide may have an improved safety profile over conventional corticosteroids because of its low [10–15%] systemic bioavailability, attributable to its extensive presystemic elimination in the gastrointestinal tract and liver.^7^ The affinity of budesonide for the glucocorticoid receptor is approximately 8.5 times higher than that of dexamethasone^8^ and 15 times higher than that of prednisolone.^9^ Because of its rapid elimination, a form of extended-release budesonide with delayed elimination was developed that uses multi-matrix [MMX^®^] technology [budesonide MMX].^10,11^ In this formulation, the active drug is embedded in an inner lipophilic [stearic acid] matrix with an overlying amphiphilic [lecithin] matrix and surrounded by a hydrophilic [hydroxypropylcellulose] matrix-forming polymer. The entire tablet is coated with gastro-resistant methacrylic acid copolymers that dissolve at a pH ≥ 7,^7,11^ thus targeting release of > 95% of active budesonide throughout the entire colon.^7^ The efficacy and safety of budesonide MMX has been reported in two phase III, randomised, placebo-controlled studies. In the CORE I^12^ and CORE II^13^ studies, budesonide MMX 9mg was significantly more efficacious than placebo for the induction of clinical and endoscopic remission and symptom resolution [ie achievement of Ulcerative Colitis Disease Activity Index [UCDAI] rectal bleeding and stool frequency subscale scores of 0] in patients with mild to moderate UC. Given the potential safety concerns associated with the use of corticosteroids, the objective of the current analysis was to conduct a safety and tolerability assessment of budesonide MMX for the induction of remission in patients with mild to moderate UC, by pooling data from five clinical studies.

## 2. Materials and Methods

### 2.1. Patients and study design

The safety data from five clinical trials were pooled for analysis: two phase III, randomised, double-blind, placebo-controlled studies (CORE I [NCT00679432] and CORE II [NCT00679380]),^12,13^ two phase II, randomised, double-blind, placebo-controlled studies [CB-01-02/05 and CRO-03-53], and one phase III open-label study [CB-01-02/06; NCT01100112]. Analyses were conducted with regard to short-term induction of remission of mild to moderate UC in patients aged ≥ 18 years [study NCT01100112 enrolled patients aged ≥ 26 years] [Table 1]. All patients receiving ≥ 1 dose of budesonide MMX in the five studies were included in the analysis.

**Table 1.** Clinical studies of budesonide MMX included in pooled safety analysis.

Study	Phase	Duration of treatment, weeks	Randomised, double-blind studies, *n* [%]				Open-label studies, *n* [%]
			Budesonide MMX 9mg/d [*n* = 288]	Budesonide MMX 6mg/d [*n* = 254]	Budesonide MMX 3mg/d [*n* = 17]	Placebo [*n* = 293]	Budesonide MMX 9mg/d [*n* = 89]
CORE I^a,^ ^12 ^	III	8	127 [44.1]	126 [49.6]	0	129 [44.0]	
CORE II^b,^ ^13 ^	III	8	128 [44.4]	128 [50.4]	0	129 [44.0]	
CB-01-02/05	II	8	15 [5.2]	0	17 [100.0]	17 [5.8]	
CB-01-02/06^c,d^	III	8					60 [67.4]
CRO-03-53	II						
Period 1		4	18 [6.3]			18 [6.1]	
Period 2		4					29 [32.6]

d, day.

^a^NCT00679432.

^b^NCT00679380.

^c^Patients may have received budesonide MMX in the CORE I study.

^d^NCT01100112.

The two phase III, randomised, double-blind studies for induction of UC remission [ie CORE I and CORE II] were identical in study design with the exception of the active reference arms (Asacol^®^ [Warner Chilcott, Rockaway, NJ, USA] in CORE I and Entocort^®^ EC [AstraZeneca LP, Wilmington, DE, USA] in CORE II).^12,13^ Briefly, patients with a UCDAI score of ≥ 4 and ≤ 10 and histological evidence of active UC received budesonide MMX 9mg, budesonide MMX 6mg, placebo, or reference medication once daily for 8 weeks.^12,13^ Remission, the primary endpoint, was strictly defined as a total UCDAI score ≤ 1, with a rectal bleeding score of 0, stool frequency score of 0, mucosal appearance score of 0 on full colonoscopy [no sign of mucosal friability], and a ≥ 1-point reduction in baseline endoscopic index score at Week 8.

One of the phase II, randomised, double-blind studies [CB-01-02/05] was a pilot study in which patients with mild to moderate UC [UCDAI score of ≥ 4 and ≤ 10], received budesonide MMX 9mg, budesonide MMX 3mg, or placebo once daily for 8 weeks. A second phase II study [CRO-03-53] consisted of two periods: a randomised, double-blind phase in which patients with mild to moderate left-sided UC [Clinical Activity Index < 14] received budesonide MMX 9mg or placebo once daily for 4 weeks [period 1], followed by an open-label phase of budesonide MMX 9mg once daily for 4 weeks [period 2]. Lastly, patients who failed to achieve remission in the CORE I study were eligible to enrol in the phase III, open-label study of budesonide MMX 9mg once daily for 8 weeks.

Adherence to ethical principles was confirmed via approval of clinical protocols by institutional review boards or ethics committees, and study conduct complied with principles of the International Conference on Harmonisation Good Clinical Practice guidelines, the Declaration of Helsinki, and national and/or local regulations relevant to research in humans at participating research sites. All patients provided written informed consent.

### 2.2. Safety assessments

Safety parameters assessed in the studies included overall adverse events [AEs], AEs resulting in study discontinuation, serious AEs [SAEs], mean morning plasma cortisol concentrations, and systemic glucocorticoid-related adverse effects. Blood samples for determination of cortisol concentrations were collected at screening, Day 1, Day 14, Day 28, and final visit [Day 56 or early withdrawal] for the CORE I and II studies; at screening, Day 0, Day 28, and final visit [Day 56 or early withdrawal] for study CB-01-02/05; at screening, Day 1, Day 28, and final visit [Day 56 or early withdrawal for study CB-01-02/06; and at screening, Day 28, and Day 56 for study CRO-03-53. AEs potentially related to the use of glucocorticoids were prespecified [ie acne, insomnia, hirsutism, flushing, fluid retention, mood changes, moon face, sleep changes, and striae rubrae] in four studies and were evaluated by investigators at screening, Day 28, final visit, and at follow-up [2 weeks after final visit] for studies CORE I and II; at screening, Day 28, Day 56, and at follow-up [~ 2 weeks after final visit] in study 01-02/05; and on Day 1, Day 28, and final visit [Day 56 or early withdrawal] in study CB-01-02/06.

### 2.3. Statistical analyses

The safety population included all patients who received ≥ 1 dose of study drug. Descriptive statistics were used to summarise demographic and baseline disease characteristics of the safety population and safety variables. Qualitative variables were presented as category counts and percentages. Statistical comparisons of safety endpoints were performed using a Fisher’s exact test, and comparisons of mean morning cortisol concentrations were performed using a t-test. Compliance was evaluated by tablet counts and calculated using the following formula:

$$\%\;compliance\;=\frac{\left(\begin{array}{l} number\;of\;tablets\;dispensed\;–\;\\ number\;of\;tablets\;returned \end{array}\right)}{\left(\begin{array}{l} study\;drug\;return\;day\;–\;\\ study\;drug\;dispense\;day \end{array}\right)}\times \;100$$

Patients were considered compliant if they took > 80% of study drug. To maintain consistency with data analysis, data collected during the last visit for patients who discontinued the study early were mapped to the final visit time point.

## 3. Results

### 3.1. Patients

Overall, 648 patients received budesonide MMX 9mg, budesonide MMX 6mg, or budesonide MMX 3mg, with 58.2% receiving budesonide MMX 9mg [Table 1]; 293 patients received placebo. Demographic and baseline disease characteristics were generally comparable across treatment groups for the randomised, controlled trials [Table 2]. The percentage of patients with proctosigmoiditis, left-sided colitis, and extensive UC were comparable within each group and among groups, except for patients in the budesonide MMX 3mg group, for whom these data were not collected. The majority of patients had moderate disease [UCDAI score 6–10] at baseline.

**Table 2.** Demographics and baseline characteristics.

Characteristic	Randomised, double-blind studies				Open-label studies
	Budesonide MMX 9mg/d^a^ [*n* = 288]	Budesonide MMX 6mg/d [*n* = 254]	Budesonide MMX 3mg/d [*n* = 17]	Placebo^a^ [*n* = 293]	Budesonide MMX 9mg/d^a^ [*n* = 89]
Age, years, mean [SD, range]	42.7 [13.0, 18–69]	43.5 [13.5, 18–75]	44.6 [14.8, 26–66]	44.0 [13.4, 18–77]	41.6 [11.6, 19–65]
Sex, *n* [%]					
Male	167 [58.0]	130 [51.2]	8 [47.1]	170 [58.0]	58 [65.2]
Female	121 [42.0]	124 [48.8]	9 [52.9]	31 [34.8]	31 [65.2]
Race, *n* [%]					
White	221 [76.7]	190 [74.8]	17 [100]	229 [78.2]	29 [32.6]
Asian	47 [16.3]	46 [18.1]	0	44 [15.0]	60 [67.4]
Black	9 [3.1]	10 [3.9]	0	8 [2.7]	0
Hispanic or Latino	8 [2.8]	7 [2.8]	0	10 [3.4]	0
Other	3 [1.0]	1 [0.4]	0	2 [0.7]	0
Duration of disease, years, mean [SD]	5.6 [6.8]	6.3 [7.3]	5.3 [7.5]	6.0 [7.2]	2.5 [2.5]
Extent of disease, *n* [%]					
Proctosigmoiditis	93 [36.5]	88 [34.6]	0	109 [42.2]	15 [25.0]
Left-sided UC	71 [27.8]	83 [32.7]	0	79 [30.6]	23 [38.3]
Extensive/pancolitis	88 [34.5]	80 [31.5]	0	63 [24.4]	22 [36.7]
Missing	3 [1.2]	3 [1.2]	17 [100]	7 [2.7]	0
Severity of disease,^b^ *n* [%]					
Mild [UCDAI 4–5]	68 [25.2]	69 [27.2]	6 [35.3]	82 [29.8]	18 [30.0]
Moderate [UCDAI 6–10]	169 [62.6]	164 [64.6]	11 [64.7]	158 [57.5]	36 [60.0]
UCDAI < 4 or > 10	22 [8.1]	10 [3.9]	0	14 [5.1]	2 [3.3]
Missing	11 [4.1]	11 [4.3]	0	21 [7.6]	4 [6.7]
UCDAI score, mean [SD, range]	6.3 [2.0, 2–10]	6.5 [1.9, 2–11]	6.1 [1.3, 4–8]	6.3 [2.0, 1–11]	6.4 [1.8, 3–10]
Endoscopic Index score, mean [SD, range]	7.1 [1.9, 1–12]	7.4 [1.9, 1–12]	6.2 [1.3, 3–7]	7.0 [2.0, 0–12]	7.2 [1.6, 3–10]
Concomitant 5-ASA use, *n* [%]	55 [19.1]	37 [14.6]	2 [11.8]	43 [14.7]	37 [41.6]

SD, standard deviation; d, day; UC, ulcerative colitis; UCDAI, Ulcerative Colitis Disease Activity Index.

^a^Baseline disease characteristics were not collected for patients in studies CB-01-02/05 [*n* = 32] and CRO-03-53 open-label period [period 2] [*n* = 29].

^b^Severity of disease based on UCDAI diary score in CORE I and II studies, and on investigator UCDAI score in study CB-01-02/05.

Overall exposure to the study drug was similar among treatment groups in the randomised, double-blind studies [Table 3]. The median duration of study drug exposure for patients receiving budesonide MMX 9mg, 6mg, 3mg, or placebo was 56.0 days for each group in the randomised, double-blind studies, and 55.0 days for patients receiving open-label budesonide MMX 9mg in the open-label studies. Patients receiving budesonide MMX 9mg in the open-label study had previously completed 8 weeks of treatment with budesonide MMX 9mg, budesonide MMX 6mg, 5-ASA 2.4g/day, or placebo, in the CORE I study. Thus, patients who received budesonide MMX 9mg in the CORE I study had 16 weeks of consecutive exposure to budesonide MMX.

**Table 3.** Summary of study drug exposure.

Duration of exposure,^a^ *n* [%]	Randomised, double-blind studies				Open-label studies
	Budesonide MMX 9mg/d [*n* = 288]	Budesonide MMX 6mg/d [*n* = 254]	Budesonide MMX 3mg/d [*n* = 17]	Placebo [*n* = 293]	Budesonide MMX 9mg/d^b^ [*n* = 89]
Mean [SD]	48.6 [15.9]	47.0 [18.0]	53.1 [9.5]	48.0 [28.3]	45.3 [15.7]
Median [range]	56.0 [1–106]	56.0 [3–89]	56.0 [28–59]	56.0 [3–421]	55.0 [8–62]
Missing	16	9	0	16	4

SD, standard deviation; d, day.

^a^Duration of exposure calculated by: [day study drug returned] – [day study drug dispensed].

^b^Patients receiving budesonide MMX 9mg in the open-label study had previously completed 8 weeks of treatment with budesonide MMX 9mg, budesonide 6mg, 5-ASA 2.4g/day, or placebo, in the CORE I study. Thus, patients who received budesonide MMX 9mg in the CORE I study had 16 weeks of consecutive exposure to budesonide MMX.

Compliance [ie ≥ 80% of doses taken] with tablets was 87.2%, 92.9%, and 85.3% for patients receiving budesonide MMX 9mg, budesonide MMX 6mg, or placebo, respectively, in the randomized, double-blind studies, and 92.1% for patients receiving budesonide MMX 9mg in the open-label studies. The majority of patients receiving budesonide MMX 9mg, 6mg, 3mg, or placebo completed the randomised, controlled studies [72.6%, 68.9%, 88.2%, and 71.7%, respectively]; 87.6% of patients receiving budesonide MMX 9mg completed the open-label studies.

The most common reason for study discontinuation in the randomised, controlled studies was treatment failure. Study discontinuation due to AEs occurred in 3.1%, 3.1%, 0%, and 3.8% of patients receiving budesonide MMX 9mg, 6mg, 3mg, and placebo, respectively, in the, double-blind studies; 2.2% of patients receiving budesonide MMX 9mg in the open-label studies discontinued on account of AEs.

### 3.2. Adverse events

The percentage of patients with any AE was generally similar among the budesonide MMX 9mg and 6mg groups [54.5% vs 60.6%, respectively] and placebo [50.5%] in the randomised, double-blind studies, and in the budesonide MMX 9mg group of the open-label studies [50.6%; Table 4]. Patients receiving budesonide MMX 3mg reported the fewest AEs [35.3%]. The frequency of drug-related AEs was similar among the budesonide MMX 9mg, 6mg, and placebo groups in the randomised, double-blind studies; patients receiving budesonide MMX 3mg or budesonide MMX 9mg in the open-label studies had the fewest drug-related AEs. The most frequently reported AEs were UC, headache, and nausea in patients receiving budesonide MMX 9mg, 6mg, or placebo in the randomised, double-blind studies, whereas decreased blood cortisol concentrations and urinary tract infection were the most commonly reported AEs in the open-label studies of budesonide MMX 9mg. The percentage of patients with infections was generally similar among the budesonide MMX 9mg, 6mg, and placebo groups [12.7%, 12.2%, and 8.5%, respectively] across randomised and open-label studies, with 5.9% of patients receiving budesonide MMX 3mg developing an infection.

**Table 4.** Summary of adverse events.

AE, *n* [%]	Randomised, double-blind studies				Open-label studies
	Budesonide MMX 9mg/d [*n* = 288]	Budesonide MMX 6mg/d [*n* = 254]	Budesonide MMX 3mg/d [*n* = 17]	Placebo [*n* = 293]	Budesonide MMX 9mg/d [*n* = 89]
Any AE	157 [54.5]	154 [60.6]	6 [35.3]	148 [50.5]	45 [50.6]
Leading to discontinuation	41 [14.2]	48 [18.9]	2 [11.8]	48 [16.4]	3 [3.4]
Treatment-related AEs^a^	78 [27.1]	63 [24.8]	2 [11.8]	70 [23.9]	12 [13.5]
Any SAE^b^	7 [2.4]	5 [2.0]	1 [5.9]	8 [2.7]	2 [2.2]
Leading to discontinuation^c^	6 [2.1]	4 [1.6]	0	4 [1.4]	1 [1.1]
AE severity					
Mild	62 [21.5]	69 [27.2]	3 [17.6]	53 [18.1]	35 [39.3]
Moderate	74 [25.7]	67 [26.4]	3 [17.6]	71 [24.2]	7 [7.9]
Severe	21 [7.3]	17 [6.7]	0	22 [7.5]	3 [3.4]
AEs reported in ≥ 4% of patients in any group^d^					
Ulcerative colitis	36 [12.5]	42 [16.5]	1 [5.9]	38 [13.0]	1 [1.1]
Headache	34 [11.8]	37 [14.6]	1 [5.9]	28 [9.6]	0
Nausea	13 [4.5]	12 [4.7]	0	12 [4.1]	1 [1.1]
Decreased blood cortisol concentrations	12 [4.2]	6 [2.4]	0	1 [0.3]	9 [10.1]
Abdominal pain	10 [3.5]	7 [2.8]	1 [5.9]	18 [6.1]	1 [1.1]
Insomnia	7 [2.4]	9 [3.5]	0	12 [4.1]	0
Pyrexia	6 [2.1]	6 [2.4]	0	12 [4.1]	0
Urinary tract infection	6 [2.1]	1 [0.4]	0	1 [0.3]	7 [7.9]
Anaemia	5 [1.7]	4 [1.6]	1 [5.9]	5 [1.7]	0
Diarrhoea	4 [1.4]	7 [2.8]	0	11 [3.8]	4 [4.5]
Nasopharyngitis	4 [1.4]	13 [5.1]	0	6 [2.0]	3 [3.4]
Asthenia	2 [0.7]	5 [2.0]	1 [5.9]	1 [0.3]	0
Haematochezia	1 [0.3]	0	1 [5.9]	4 [1.4]	1 [1.1]
Nephrolithiasis	1 [0.3]	0	1 [5.9]	0	0
Acute tonsillitis	0	0	1 [5.9]	2 [0.7]	0
Fluid retention	0	2 [0.8]	1 [5.9]	3 [1.0]	1 [1.1]

AE, adverse event; SAE, severe adverse event; d, day.

^a^ *p* = 0.26 for budesonide MMX 9mg vs budesonide MMX 3mg and *p* = 0.56 for budesonide MMX 9mg vs budesonide MMX 6mg [randomised, double-blind studies].

^b^ *p* = 0.37 for budesonide MMX 9mg vs budesonide MMX 3mg and *p* = 0.78 for budesonide MMX 9mg vs budesonide MMX 6mg [randomised, double-blind studies].

^c^ *p* > 0.99 for budesonide MMX 9mg vs budesonide MMX 3mg and *p* = 0.76 for budesonide MMX 9mg vs budesonide MMX 6mg [randomised, double-blind studies].

^d^AEs presented in descending order of frequency for budesonide MMX 9mg group, then alphabetically for AEs with equal frequency.

Most AEs were mild [24%] or moderate [24%] in intensity; 7% of AEs were considered by investigators to be severe. In the randomised, double-blind studies, UC was the most commonly reported SAE in patients receiving budesonide MMX 9mg, 6mg, or placebo [3.1%, 3.9%, or 3.1%, respectively]. No SAEs were reported by patients receiving budesonide MMX 3mg. In the open-label studies, SAEs were reported by 3.4% of patients, and included defaecation urgency, oral abscess, and endometrial hyperplasia.

Overall, SAEs were reported by 2.4% of patients across all treatment groups; the percentage of patients with SAEs was similar across groups [Table 4]. Serious AEs led to study discontinuation in 1.9%, 1.6%, and 1.4% of patients receiving budesonide MMX 9mg, 6mg, or placebo, respectively, across randomised and open-label studies. No patient receiving budesonide MMX 3mg experienced an SAE leading to study discontinuation. UC-related events [eg worsening of UC] were the most common SAE leading to withdrawal in patients receiving budesonide MMX 9mg, 6mg, or placebo. No deaths were reported.

### 3.3. Plasma cortisol concentrations

**Figure 1.**
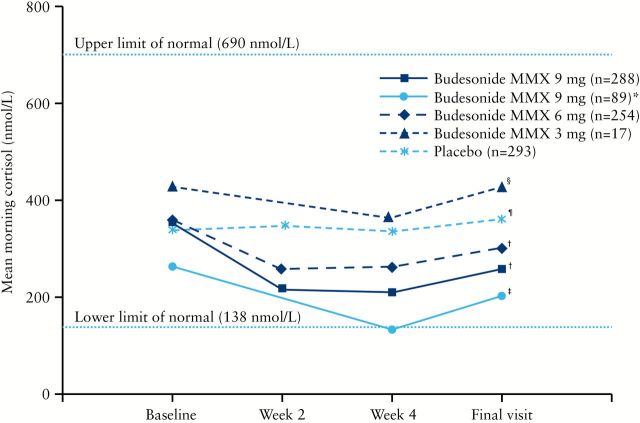
Mean morning plasma cortisol concentrations. *Of the 89 patients who received open-label budesonide MMX 9mg, several had previously completed 8 weeks of treatment with budesonide MMX 9mg [*n* = 12] or budesonide MMX 6mg [*n* = 16] in CORE 1, and thus had 16 weeks of consecutive exposure to budesonide MMX. ^†^ *p* < 0.0001; ^‡^ *p* = 0.03; ^§^ *p* = 0.93; ^¶^ *p* = 0.01, for changes from baseline to final visit.

In the randomised, double-blind studies, mean morning plasma cortisol concentrations decreased in a dose-dependent manner from baseline to final visit for budesonide MMX 9mg, 6mg, and 3mg [19.4%, 10.0%, and 1.0%, respectively] and generally remained within normal limits [ie 138–690 nmol/l] during the study [Figure 1]. Mean morning plasma cortisol concentrations at baseline decreased in patients receiving budesonide MMX 9mg in the open-label studies compared with patients in the randomised, controlled studies. Further, patients receiving budesonide MMX 9mg had mean morning plasma cortisol concentrations of 129.9 nmol/l at Week 4; this was below normal concentrations, but mean cortisol concentrations returned to normal concentrations at the final visit. The changes from baseline to final visit were statistically significant for placebo [increase from baseline] and all budesonide MMX groups [decrease from baseline] except budesonide MMX 3mg.

### 3.4. Potential glucocorticoid-related adverse effects

The incidence of potential glucocorticoid-related adverse effects was < 10% across all treatment groups [Table 5]. There was no apparent dose-related increase in potential glucocorticoid-related adverse effects following treatment with budesonide MMX. Further, the incidence of potential glucocorticoid-related adverse effects was similar when mean morning cortisol concentrations at final visit were distributed across quartiles. When patients with low morning plasma cortisol concentrations [< 138 nmol/l] at the final visit were selectively examined, mood and sleep changes were the most frequently reported potential glucocorticoid-related adverse effects among patients who received budesonide MMX 9mg, 6mg, or placebo [Table 6]. In the budesonide MMX 3mg group, one patient reported fluid retention.

**Table 5.** Summary of potential glucocorticoid-related adverse effects.

Effect, *n* [%]	Randomised, double-blind studies				Open-label studies
	Budesonide MMX 9mg/d [*n* = 270]^a^	Budesonide MMX 6mg/d [*n* = 254]	Budesonide MMX 3mg/d [*n* = 17]	Placebo [*n* = 293]	Budesonide MMX 9mg/d [*n* = 60]^a^
Any potential glucocorticoid-related adverse effect^b,c^	26 [9.6]	19 [7.5]	1 [5.9]	27 [9.8]	5 [8.3]
Mood changes	9 [3.3]	10 [3.9]	0	11 [4.0]	0
Sleep changes	7 [2.6]	10 [3.9]	0	12 [4.4]	0
Acne	6 [2.2]	2 [0.8]	0	5 [1.8]	1 [1.7]
Insomnia	6 [2.2]	6 [2.4]	0	8 [2.9]	1 [1.7]
Moon face	3 [1.1]	3 [1.2]	0	4 [1.5]	3 [5.0]
Fluid retention	2 [0.7]	3 [1.2]	1 [5.9]	3 [1.1]	1 [1.7]
Hirsutism	1 [0.4]	0	0	0	0
Flushing	0	1 [0.4]	0	3 [1.1]	0
Striae rubrae	0	0	0	2 [0.7]	0

d, day.

^a^Adverse events potentially related to the use of glucocorticoids were not prespecified in study CRO-03-53 and thus this population was not included in the analysis.

^b^Potential glucocorticoid-related adverse effects presented in descending order of frequency for budesonide MMX 9mg group, then alphabetically for AEs with equal frequency.

^c^ *p* > 1.0 for budesonide MMX 9mg vs budesonide MMX 3mg [randomised, double-blind studies].

**Table 6.** Summary of potential glucocorticoid-related adverse effects in patients with low cortisol morning plasma concentrations [< 138 nmol/l] at final visit.^a^

Effect, *n* [%]	Budesonide MMX 9mg/d [*n* = 63]	Budesonide MMX 6mg/d [*n* = 47]	Budesonide MMX 3mg/d [*n* = 2]	Placebo [*n* = 8]
Any glucocorticoid-related adverse effect^b^	9 [14.3]	2 [4.3]	1 [50.0]	2 [25.0]
Mood changes	4 [6.3]	2 [4.3]	0	1 [12.5]
Acne	2 [3.2]	0	0	0
Fluid retention	1 [1.6]	0	1 [50.0]	0
Hirsutism	1 [1.6]	0	0	0
Insomnia	1 [1.6]	1 [2.1]	0	0
Moon face	1 [1.6]	0	0	0
Sleep changes	1 [1.6]	1 [2.1]	0	2 [25.0]
Flushing	0	0	0	0
Striae rubrae	0	0	0	0

d, day.

^a^Patients in randomised, double-blind, phase II and III studies.

^b^Potential glucocorticoid-related adverse effects presented in descending order of frequency for budesonide MMX 9mg group, then alphabetically for AEs with equal frequency.

## 4. Discussion

Budesonide MMX is a second-generation oral corticosteroid indicated for the induction of remission in patients with active, mild to moderate UC.^10^ In a pooled safety analysis of five clinical studies, budesonide MMX administered for up to 8 weeks demonstrated a favourable safety and tolerability profile for the induction of remission in patients with active, mild to moderate UC. The overall AE profile in patients taking budesonide MMX was comparable to that of those taking placebo. Pooling of safety data for 648 patients receiving budesonide MMX 9mg, 6mg, or 3mg provided a more robust evaluation of the safety of budesonide MMX and allowed for greater accuracy in estimating the incidence of drug-related AEs, especially when compared with assessments of the studies individually. The findings of this pooled analysis support the short-term safety and tolerability of budesonide MMX for the induction of remission in patients with mild to moderate UC.

The frequency and intensity of AEs were generally comparable across treatment groups, with the fewest AEs reported in the budesonide MMX 3mg group; however, this treatment group also included the fewest patients [*n* = 17]. Suppression of the immune system may occur during treatment with systemic corticosteroids,^6^ thus increasing the risk of infection in patients. However, this analysis suggested no apparent dose-related increase in the incidence of infections. Further, patients receiving budesonide MMX 9mg or 6mg had a rate of infection similar to that of patients receiving placebo, and patients receiving budesonide MMX 3mg had the lowest incidence of infection. The occurrence of SAEs during treatment with budesonide MMX was infrequent, with patients in randomised, double-blind studies in the budesonide MMX 9mg group reporting the greatest frequency of SAEs [2.4%]. The AE profile of patients in this pooled safety analysis is comparable to previous reports of patients with left-sided UC or Crohn’s disease who received once-daily oral budesonide 9mg for 8 to 16 weeks.^14–16^

Decreased plasma cortisol concentrations and glucocorticoid-related adverse effects [e.g. moon face, striae rubrae, mood changes, sleep changes] are associated with treatment with systemic corticosteroids.^17–19^ However, in the current study, although the changes from baseline to final visit were statistically significant for placebo and all budesonide MMX groups except budesonide MMX 3mg, morning plasma cortisol concentrations remained within normal concentrations [ie 138–690 nmol/l] for the majority of patients. Mean cortisol concentrations decreased to 129.9 nmol/l after 4 weeks in patients receiving open-label treatment with budesonide MMX 9mg, but recovered to normal concentrations [ie 200.1 nmol/l] by the final visit. This finding was consistent with results of a previous study of once-daily oral budesonide 15mg, 9mg, or 3mg in patients with Crohn’s disease,^20^ which showed that patients receiving oral budesonide 9mg had a decrease in median morning cortisol concentrations to 135.2 nmol/l after 4 weeks, that returned to normal after 8 weeks. Mean cortisol concentrations after 4 weeks following treatment with budesonide MMX 9mg in the open-label studies were decreased compared with those for patients who received budesonide MMX 9mg in the randomised, controlled studies; this finding may be due to the number of patients who had previously completed 8 weeks of treatment with budesonide MMX 9mg or 6mg in the CORE I study. Results of this analysis suggest that short-term [up to 8 weeks] treatment with budesonide MMX had a minimal effect on the hypothalamic-pituitary-adrenal axis, a finding that is consistent with the pharmacodynamic activity of budesonide. The minimal systemic exposure of budesonide is due to its > 90% first-pass hepatic metabolism.^21^ Glucocorticoid-related adverse effects occurred in < 10% of patients in each treatment group. Patients with low [< 138 nmol/l] plasma cortisol concentrations at final visit did not appear to be at increased risk for potential glucocorticoid-related adverse effects compared with placebo. However, it is difficult to draw firm conclusions given the small number of patients in the budesonide MMX 3mg and placebo groups.

Several limitations of this analysis should be noted, including differences in study design among the clinical trials analysed [eg randomised, controlled studies; open-label studies] and the wide variation in the number of patients across budesonide MMX treatment groups [eg budesonide MMX 3mg, *n* = 17; budesonide MMX 6mg, *n* = 254; vs budesonide MMX 9mg, *n* = 377]. Further, the studies were not designed, and thus not sufficiently powered, to specifically evaluate safety outcomes. Although the studies included did not contribute equally to the analysis, it is reassuring that a low incidence of AEs were reported in the two large, randomised, controlled trials.

In conclusion, the findings of this pooled safety analysis demonstrate that short-term treatment with budesonide MMX for the induction of remission in patients with mild to moderate UC was well tolerated, with an AE profile comparable to placebo. Although 5-ASAs are currently the most frequently prescribed treatment for the induction of remission of mild to moderate UC,^4,5^ budesonide MMX is a second-generation glucocorticosteroid indicated for the induction of remission in patients with active, mild to moderate ulcerative colitis and may be an alternative to conventional corticosteroid in such patients.

## Conflicts of Interest

G Lichtenstein has received research grants and/or has served as a consultant for Abbott Corp., Alaven, Bristol-Myers Squibb, Janssen, Elan Pharmaceuticals, Ferring Pharmaceuticals, Hospira, Luitpold Pharmaceuticals/American Regent, Meda Pharmaceuticals, Merck/Schering-Plough, Millennium Pharmaceuticals, Ono Pharmaceutical Co., Ltd, Pfizer, Proctor and Gamble, Prometheus Laboratories, Salix, a Division of Valeant Pharmaceuticals North America LLC, Bridgewater, NJ, USA, Santarus, Inc. [previously a wholly owned subsidiary of Salix Pharmaceuticals, Inc.], Shire, UCB, Warner Chilcott, and Wyeth.

S Travis received consulting fees from AbbVie, Asahi-Kasei, Bristol-Myers Squibb, Coronado Biosciences, Cosmo Technologies Ltd, Ferring Pharmaceuticals, Genentech, Genzyme Corp., GlaxoSmithKline, Janssen, Lexicon Pharmaceuticals, Merck Research Laboratories, Millennium Pharmaceuticals, Nisshin Kyorin Pharmaceutical Co., Ltd, Novartis, Novo Nordisk A/S, NPS Pharmaceuticals, PDL BioPharma, Pfizer, Procter and Gamble, Santarus, Inc., Schering Plough, Shire, Sigmoid Pharma Ltd, Tillotts Pharma AG, TxCell SA, UCB, and Warner Chilcott UK Ltd; he has received research grants from AbbVie, Genentech, GlaxoSmithKline, Janssen, Novartis, Pfizer, Procter and Gamble, Shire, and UCB; and he has received payments for lectures/speakers’ bureau participation from AbbVie, Ferring Pharmaceuticals, Janssen, and Warner Chilcott UK Ltd.

S. Danese has served as a speaker, consultant and advisory board member for Abbott Laboratories, Actelion, Alpha Wassermann, Astra Zeneca, Cellerix SA, Cosmo Pharmaceuticals S.p.A., Ferring Pharmaceuticals, Genentech, Grünenthal Group, Johnson & Johnson, Merck & Co, Millennium Pharmaceuticals, Novo Nordisk A/S, Nycomed, Pfizer, Pharmacosmos A/S, Salix, Schering-Plough, Takeda Pharmaceutical Co., Ltd, UCB, and Vifor Pharma.

G D’Haens has served as a speaker, consultant, and/or principal investigator for AbbVie, AM-Pharma BV, Amgen, AstraZeneca, Boehringer Ingelheim, Centocor Ortho Biotech, Cosmo Pharmaceuticals S.p.A, Dr Falk Pharma GmbH, Elan Pharmaceuticals, Ferring Pharmaceuticals, Galapagos NV, Giuliani S.p.A, Given Imaging, GlaxoSmithKline, Janssen, Millennium Pharmaceuticals, MSD, Norgine, Novo Nordisk A/S, Otsuka Pharmaceutical, PDL BioPharma, Pfizer, PhotoPill Medical Ltd, Shire, Takeda Pharmaceutical Co., Ltd, Teva Pharmaceutical Industries, Ltd, Tillotts Pharma AG, UCB, Versant Ventures, and Vifor Pharma.

L Moro is an employee of Cosmo Technologies Ltd, a subsidiary of Cosmo Pharmaceuticals S.p.A., and owns stock in Cosmo Pharmaceuticals S.p.A.

R Jones is an employee of Cosmo Technologies Ltd, a subsidiary of Cosmo Pharmaceuticals S.p.A., and owns stock in Cosmo Pharmaceuticals S.p.A.

M Huang is a former employee of Santarus, Inc.

E D Ballard is a former employee of Santarus, Inc.

R Bagin is a former employee of Santarus, Inc.

Y Hardiman is a former employee of Santarus, Inc.

R Harris-Collazo is a former employee of Santarus, Inc.

W Sandborn has served as a consultant for AbbVie, ActoGeniX NV, AGI Therapeutics, plc, Alba Therapeutics Corp., Albireo, Alfa Wassermann, AM-Pharma BV, Amgen, Anaphore, Aptalis, Astellas, Athersys, Inc., Atlantic Healthcare plc., BioBalance Corp., Boehringer-Ingelheim, Bristol-Myers Squibb, Celek Pharmaceuticals, Celgene, Cellerix SA, Cerimon Pharmaceuticals, ChemoCentryx, CoMentis, Coronado Biosciences, Cosmo Technologies Ltd, Cytokine Pharmasciences, Eagle Pharmaceuticals, Eisai Medical Research Inc., Elan Pharmaceuticals, Eli Lilly, EnGene, Inc., Enteromedics, Exagen Diagnostics, Inc., Ferring Pharmaceuticals, Flexion Therapeutics, Inc., Funxional Therapeutics Ltd., Genentech, Genzyme Corp., Gilead Sciences, Given Imaging, GlaxoSmithKline, Human Genome Sciences, Ironwood Pharmaceuticals, Janssen, KaloBios Pharmaceuticals, Inc., Lexicon Pharmaceuticals, Lycera Corporation, Meda Pharmaceuticals, Merck & Co., Merck Research Laboratories, MerckSerono, Millennium Pharmaceuticals, Nisshin Kyorin Pharmaceutical Co., Ltd, Novo Nordisk A/S, NPS Pharmaceuticals, Optimer Pharmaceuticals, Orexigen Therapeutics, Inc., PDL BioPharma, Pfizer, Procter and Gamble, Prometheus Laboratories, ProtAb Ltd, Purgenesis Technologies, Inc., Receptos, Relypsa, Inc., S.L.A. Pharma [UK] Ltd, Salient Pharmaceuticals, Salix, Santarus, Inc., Shire, Sigmoid Pharma Ltd, Sirtris Pharmaceuticals, Inc., Targacept, Teva Pharmaceutical Industries, Ltd, Therakos, Tillotts Pharma AG, TxCell SA, UCB, Vascular Biogenics Ltd, Viamet Pharmaceuticals, and Warner Chilcott UK Ltd. he has received speakers’ bureau fees for AbbVie, Bristol-Myers Squibb, and Janssen; and he has received financial support for research from AbbVie, Bristol-Myers Squibb, Genentech, GlaxoSmithKline, Janssen, Millennium Pharmaceuticals, Novartis, Pfizer, Procter and Gamble, Shire, and UCB.

## Funding

The clinical trials included in this analysis were funded by Cosmo Pharmaceuticals, S.p.A., and Santarus, Inc., and this current analysis was funded by Santarus, Inc. Funding to pay Open Access publication charges for this article was provided by Valeant Pharmaceuticals North America LLC, Bridgewater, NJ, USA.

## Author Contributions

G Lichtenstein contributed to the design of the studies and collection and analysis of data, interpretation of the data, and writing the manuscript [many comments on drafts], and provided significant advice or consultation. S Travis contributed to the concept, design, and conduct of the experiment, analysis/interpretation of the data, and writing the manuscript [many comments on drafts], and provided significant advice or consultation. S Danese contributed to the concept, design, and conduct of the experiment, and analysis/interpretation of the data, and provided significant advice or consultation. G D’Haens contributed to the conduct of the experiment, collection of data, and manuscript review, and provided significant advice or consultation. L Moro contributed to the concept, design, and conduct of the experiment, analysis/interpretation of the data, and writing of the manuscript, and provided significant advice or consultation. R Jones contributed to the concept, design, and conduct of the experiment and collection and analysis/interpretation of data. M Huang contributed to the analysis/interpretation of data, and writing of the manuscript, and provided significant advice or consultation. ED Ballard contributed to the concept, design, and conduct of the experiment, collection and analysis of data, and early draft manuscript writing. R Bagin contributed to the design of the studies and collection and analysis of data. Y Hardiman contributed to the analysis/interpretation of data and provided significant advice in statistical analysis. R Harris-Collazo contributed to the analysis and interpretation of data and writing of the manuscript. W Sandborn contributed to the concept, design, and conduct of the experiment, analysis/interpretation of data, and critical revision of the manuscript, and provided significant advice in statistical analysis. All authors contributed to interpretation of data and analyses and writing, critically reviewing and editing the manuscript. All authors read and approved the final manuscript.
